# The Evolution of Vascular Interventional Radiology and Endovascular Surgery: An Overview of Recent Advances

**DOI:** 10.3390/jcm14030939

**Published:** 2025-02-01

**Authors:** Ádám Szőnyi, Balázs Bence Nyárády, Luca Mezzetto, Edit Dósa

**Affiliations:** 1 Heart and Vascular Center, Semmelweis University, 1122 Budapest, Hungary; szonyi.adam@stud.semmelweis.hu (Á.S.); nyarady.balazs.bence@semmelweis.hu (B.B.N.); 2 Department of Vascular Surgery, University of Verona, 37124 Verona, Italy; luca.mezzetto@aovr.veneto.it

## 1. Introduction

Vascular interventional radiology (VIR) and endovascular surgery (EVS) are dynamic and rapidly evolving fields in modern medicine. In recent years, these two disciplines have undergone a period of substantial development, driven by innovative technologies and the continuous refinement of endovascular techniques. Interventional procedures traditionally consist of three primary phases: the pre-procedural, procedural, and post-procedural phases. In this Editorial, we aim to provide a concise review of these phases, highlighting the most significant advances in each.

## 2. Pre-Procedural Phase

A careful pre-procedural phase, characterized by judicious patient selection and systematic treatment planning, can lay the foundation for a successful intervention. A key aspect of innovation in this phase is the expanding role of artificial intelligence, which has made considerable strides in non-invasive radiology and is currently transforming VIR and EVS. Specifically, artificial intelligence models have demonstrated potential in enhancing patient selection for intervention by integrating clinical and radiological datasets, resulting in more precise and dependable predictions of patient responses to treatment [1]. The domain of artificial intelligence also offers opportunities in pre-procedural treatment planning, with tools such as (1) automatic image segmentation for more consistent and faster image analysis, (2) accurate needle trajectory assignment, and (3) advanced image processing to address issues such as motion artifacts from respiratory or bowel movements [2].

## 3. Procedural Phase

The procedural phase of vascular interventions comprises a complex workflow influenced by several factors. In this section, the focus will be on the most impactful advancements pertaining to arterial access, fusion imaging, robot-assisted interventions, and drug-coated devices in the treatment of peripheral arterial disease.

The initial step in any endovascular procedure is to allow access to the circulatory system. Over the years, various techniques have been developed to ensure optimal access while minimizing complications and reducing hospital stays. Transradial access, a conventional approach in interventional cardiology, has recently gained traction in interventional radiology due to its lower complication rates and the increasing availability of longer sheaths, wires, and catheters. Despite its clear advantages, transradial access is not without limitations, including an elevated risk of neurological complications and reduced support in distal regions owing to the length of catheter systems [3]. Nonetheless, the reliability of transradial access has been proven in procedures, including embolization, neurointerventions, and simpler proximal lower extremity interventions [4,5].

Image guidance is imperative in VIR and EVS, and state-of-the-art advancements in imaging technology have further improved its effectiveness. A notable innovation is fusion imaging, which combines images from multiple modalities (i.e., ultrasound, fluoroscopy, cone-beam CT, conventional CT, and MRI) by overlaying or displaying them side by side in a coordinated manner [6,7]. The fusion of multimodal volumetric data allows for the superior visualization of anatomical structures, blood vessels, and target lesions, facilitating target identification, device positioning, and accurate treatment delivery [8].

Robotic systems have revolutionized numerous surgical specialisms and are now entering the field of VIR and EVS. Endovascular robotic systems hold promise in relation to the more precise and stable navigation of wires and catheters while significantly reducing radiation exposure among healthcare personnel [9]. This technology is particularly exciting because it can augment device navigation in small-caliber arteries. In addition, the combination of robotic systems with artificial intelligence-based tools and fusion imaging can enhance procedural accuracy and optimize outcomes [9].

A growing body of evidence indicates that drug-eluting devices offer significant benefits in managing steno-occlusive lesions of the femoropopliteal segment, both in terms of immediate and mid-term outcomes [10,11]. These devices include drug-coated balloons and drug-eluting stents. The main advantage of these devices over bare balloons and bare metal stents is their ability to release cytostatic drugs, such as sirolimus or paclitaxel, which help to combat intimal hyperplasia and subsequent restenosis [12]. In the below-the-knee region, which is traditionally referred to as the “no metal zone”, drug-eluting bioabsorbable scaffolds represent a potential solution for the treatment of focal flow-limiting dissections by providing temporary support to the vessel wall without leaving permanent metal implants [13].

## 4. Post-Procedural Phase

Post-procedural follow-up is essential in assessing the clinical status of patients and promptly detecting recurrent symptoms. The advent of telemedicine and wearable remote monitoring platforms has empowered clinicians to manage patients more effectively by leveraging real-time data transmission. These tools also assist clinicians in ensuring that patients adhere to prescribed exercise programs, lifestyle modifications, and medication regimens [14].

## 5. Conclusions

In summary, VIR and EVS are dramatically evolving fields in which technological advances are being rapidly incorporated into everyday clinical practice. These advancements have resulted in substantial improvements in the accuracy, safety, and efficacy of endovascular therapies for a wide range of vascular pathologies.
